# Determining the Evolution of Headache Among Regular Users of a Daily Electronic Diary via a Smartphone App: Observational Study

**DOI:** 10.2196/26401

**Published:** 2021-07-07

**Authors:** Bianca Raffaelli, Jasper Mecklenburg, Lucas Hendrik Overeem, Simon Scholler, Markus A Dahlem, Tobias Kurth, Ana Sofia Oliveira Gonçalves, Uwe Reuter, Lars Neeb

**Affiliations:** 1 Department of Neurology Charité – Universitätsmedizin Berlin Berlin Germany; 2 Newsenselab GmbH Berlin Germany; 3 Institute of Public Health Charité – Universitätsmedizin Berlin Berlin Germany

**Keywords:** headache, migraine, mobile app, headache app, electronic diary, app, pain, frequency, intensity

## Abstract

**Background:**

Smartphone-based apps represent a major development in health care management. Specifically in headache care, the use of electronic headache diaries via apps has become increasingly popular. In contrast to the soaring volume of available data, scientific use of these data resources is sparse.

**Objective:**

In this analysis, we aimed to assess changes in headache and migraine frequency, headache and migraine intensity, and use of acute medication among people who showed daily use of the headache diary as implemented in the freely available basic version of the German commercial app, M-sense.

**Methods:**

The basic version of M-sense comprises an electronic headache diary, documentation of lifestyle factors with a possible impact on headaches, and evaluation of headache patterns. This analysis included all M-sense users who had entered data into the app on a daily basis for at least 7 months.

**Results:**

We analyzed data from 1545 users. Mean MHD decreased from 9.42 (SD 5.81) at baseline to 6.39 (SD 5.09) after 6 months (*P*<.001; 95% CI 2.80-3.25). MMD, AMD, and migraine intensity were also significantly reduced. Similar results were found in 985 users with episodic migraine and in 126 users with chronic migraine.

**Conclusions:**

Among regular users of an electronic headache diary, headache and migraine frequency, in addition to other headache characteristics, improved over time. The use of an electronic headache diary may support standard headache care.

## Introduction

The rapid development of modern technologies has led to major advances in health care [1,2]. The trend toward eHealth and, in particular, mobile health (mHealth) has entered the headache field and includes the use of smartphone-based apps. In recent years, headache apps have increased not just in number but also in functionality. Functions comprise headache documentation in so called e-diaries, the recording of lifestyle factors that may potentially influence headaches, and the automatic incorporation of external information, such as weather conditions. Some apps also provide digital-based education programs and self-managed nonpharmacological interventions, like relaxation methods.

Tracking headaches and associated symptoms is a pillar of headache management. Digital headache documentation has proven to have a higher compliance than do paper headache diaries [3,4]. From a clinical perspective, proper documentation can facilitate health care providers in diagnosing headache disorders, evaluating the course of the disease, and assessing treatment effects. It also provides patients with a better understanding and awareness for their headaches [5]. Through a comprehensive analysis of headache attacks and associated factors, digital algorithms can recognize and offer a clear presentation of headache patterns and may improve the self-management of headaches. Conversely, the constant dealing with headache symptoms and potential triggers may lead to an increased focus on the disease. In this way, these apps could even become stressors themselves and cause a worsening of headaches [6].

Hence, apps may offer new opportunities but also entail possible risks in the outpatient care of migraine patients [7]. Previous studies indicate that the complementary use of smartphone apps and other internet-based technologies could lead to a better treatment of headache patients compared to standard care [8]. However, evidence of positive health outcomes through the use of mHealth is scarce [9]. Most available headache apps lack scientific evaluation, and uniform quality standards have not yet been established [10,11].

In this analysis, we aimed to assess the evolution of headache characteristics among regular users of the freely available basic version of a commercial headache app (M-sense). We tested whether headache and migraine frequency, headache and migraine intensity, and days with intake of acute medication differed between baseline and after 6 months. In extension analyses, we compared outcomes at baseline to those at 12 months.

## Methods

### M-sense

M-sense is a certified German headache app developed by Newsenselab. In Germany, Austria, and Switzerland, it has been available for Android since 2016 and for iOS since 2017 [12]. From September 2016 to May 2020, the app was downloaded approximately 250,000 times and had nearly 85,000 registered users.

There are 2 versions of the app: a free “basic” version, and an additional, paid, “active” version. Our study focused on the basic version. In this version, users can document their headache information in a diary according to a predefined scheme. This includes the start and end of the headache, maximal pain intensity (on a 11-point numeric rating scale from 0 to 10), unilateral headache (yes or no), throbbing headache (yes or no), worsening through physical activity (yes or no), nausea (yes or no), vomitus (yes or no), photophobia (yes or no), phonophobia (yes or no), and migraine aura (yes or no). For each headache attack, users can record the intake of acute medication including the name and dose of the drug and time of intake. A standardized, validated algorithm based on the International Classification of Headache Disorders (ICHD-3) criteria [13] classifies single headache attacks as migraine, tension-type headache (TTH), or nonmigraine or non-TTH [14]. A detailed description of the classification algorithm can be found in the publication by Roesch et al [14]. For the purposes of this study, we assessed migraine days and headache days. A migraine day was defined as a calendar day on which the user experienced a qualified migraine attack. To identify if an attack should be counted as migraine or TTH, we applied all relevant ICHD-3 guidelines [13]. A qualified migraine attack needed to meet the following ICHD-3 criteria for migraine: a headache with or without aura lasting at least 4 hours with both features A (at least 2 of the following: unilateral location, pulsatile quality, moderate or severe pain intensity, and aggravation caused by physical activity or avoidance of physical activity) and B (during headache, at least 1 of the following: nausea and/or vomiting and/or photophobia and phonophobia). Any headache attacks accompanied by intake of a migraine-specific medication (eg, triptans) to treat a headache or accompanied by aura were defined as migraine regardless of the duration and pain features or associated symptoms. The ICHD-3 classifications of TTH and probable migraine differ depending on whether or not a patient has received a diagnosis. During the onboarding process of the app, users were asked if they received a headache diagnosis by a health care professional. In users with a previous migraine diagnosis, all headaches that fulfilled the criteria of both TTH and probable migraine were counted as migraine according to the ICHD-3. A headache day was defined as any calendar day on which the user experienced a headache attack (including migraine). The app provides an exportable, graphic overview of headache attacks, subdivided into migraine, TTH, and others.

Users can also enter daily data on predefined lifestyle factors with a possible impact on headaches (eg, sleep duration, sleep quality, or stress level). In addition, the app collects weather data. Users can see the course of these factors over time and their mean values on the days immediately preceding a headache and on headache days. After 60 days of daily entries, the app analyzes correlations between the entered factors and headaches and, if necessary, a further medical consultation is recommended.

### Population and Outcomes

This analysis included all M-sense users who entered data in the app every day for at least 7 months on a daily basis. No further inclusion or exclusion criteria were applied.

The primary outcome was the number of monthly headache days (MHD). Secondary outcomes included monthly migraine days (MMD), monthly days with intake of acute headache medication (AMD), mean monthly headache intensity, and mean monthly migraine intensity.

The outcomes were selected based on recent guidelines for migraine trials [15]. As recommended in the guidelines for trials of behavioral treatments for recurrent headache, we focused on all monthly headache days and not only on moderate or severe monthly headache days, in addition to monthly migraine days [16].

A month was defined as 4 weeks (28 days), beginning with the first day of app use. Both triptan and nontriptan pain medication (eg, nonsteroidal anti-inflammatory drugs or paracetamol) counted as acute headache medication.

We defined the first month after the app installation as baseline. We compared the primary and secondary outcome measures between the baseline phase and the sixth month after baseline (ie, the seventh month of app use).

After analyzing the entire population, we performed subgroup analyses for users with headache and migraine frequency compatible with the diagnosis of episodic or chronic migraine according to the ICHD-3 criteria [13] and based on the first 90 days of app use. Another concomitant headache diagnosis (eg, TTH in patients with episodic migraine) was possible.

Additionally, we conducted an analysis for users who continued to use the app for at least 13 months. In this population, we compared the aforementioned parameters between the baseline month and the 12th month after baseline (ie, the 13th month of app use). We further recorded the headache frequency and medication use 1, 7, and 13 months after users had begun accessing the app.

### Data Processing

For data protection reasons, Newsenselab provided a sample data set containing dummy data in the same format as the real data set. We tested our analysis code locally against the sample data set. The Newsenselab team (SS and MAD) then ran the analysis code on the real, personal data and provided the aggregated output data. Through this procedure, the research team had no access to the individual data sets but only to aggregated results for the predefined sample and outcome variables.

### Ethics

By installing the app, the users agreed to the general terms and conditions of M-sense as well as the privacy policy. These include the storage of personal and health data on an Amazon Web Services (Amazon.com, Inc) server in encrypted form and the transfer of these data in anonymous form to third parties for medical research purposes.

We used only aggregated data. In accordance with the local legislation and institutional requirements, use of aggregated data does not require institutional review board approval.

### Statistical Analysis

The statistical analysis was performed using R version 3.6.2 (The R Foundation for Statistical Computing). Demographics and monthly headache characteristics were summarized with descriptive statistical methods. For categorical variables, we report absolute frequencies and percentages. For numerical variables, we report mean and SD.

Outcome measures were compared between baseline and the last considered month using paired samples *t* tests. Due to the large sample size, calculation for normal distribution was not necessary [17]. To compare whether the headache frequencies at baseline, month 6, and month 12 differed, we used analysis of variance (ANOVA) for repeated measures. A 2-tailed *P* value ≤.05 was considered statistically significant.

## Results

### Demographics and Headache Diagnosis

**Figure 1:**
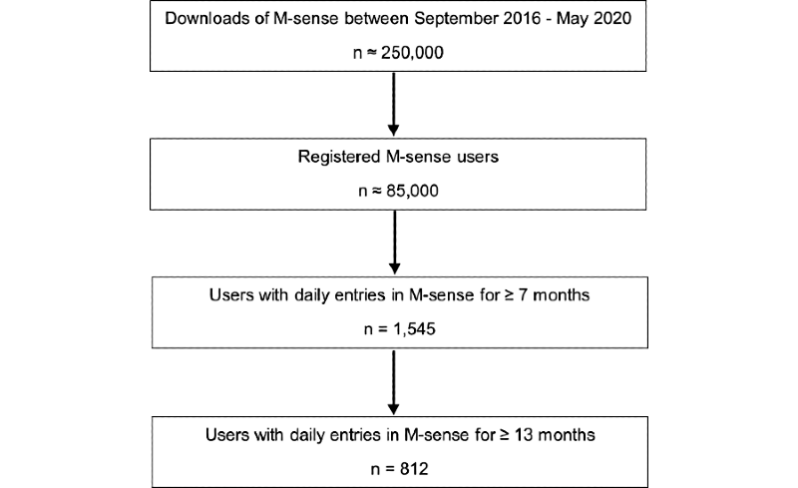
Flowchart of the user selection process.

Between September 2016 and May 2020, 1545 users recorded headache information every day for 7 months (Figure 1). Of the users with available data, most were female (920/1047, 87.87%) with a mean age of 37.2 years (SD 11.1).

The app supported the diagnosis of “episodic migraine” in 985 cases and “chronic migraine” in 126 cases. The other users reported headaches compatible with the diagnosis of TTH or a not-further-classified headache disorder. Demographic features for all patients, patients with episodic migraine, and patients with chronic migraine are shown in Table 1.

**Table 1:** Demographic features of M-sense users with daily data entries for 6 months after baseline. Percentages are calculated for the available data.

Characteristic	All users	Episodic migraine	Chronic migraine
Distribution, n	1545	985	126
Female sex, n (%)	920 (87.9)	607 (89.8)	85 (91.4)
Missing data for sex, n	498	309	33
Age (years), mean (SD)	37.2 (11.1)	37.7 (11.1)	36.7 (11.7)
Missing data for age, n	393	252	23

### Changes in Headache Characteristics Over 6 Months of App Use

**Figure 2:**
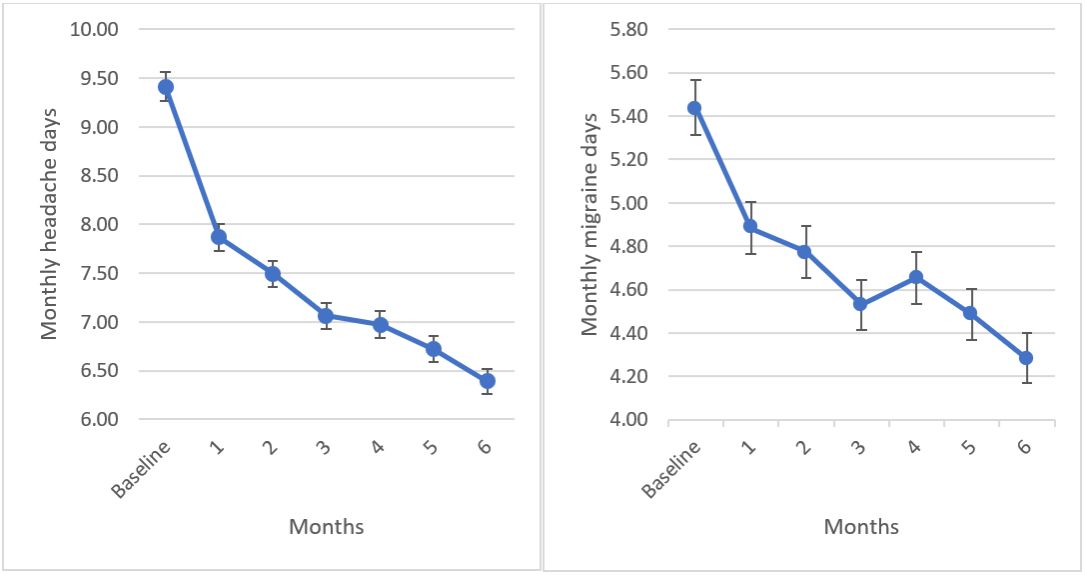
Monthly headache days and monthly migraine days (mean and SE) during baseline and in the first 6 months of M-sense use after baseline.

During the first month of use, users reported on average 9.42 MHD (SD 5.81), which decreased to 6.39 (SD 5.09) after 6 months (*P*<.001; 95% CI 2.80-3.25). MMD decreased from 5.44 (SD 4.98) during baseline to 4.28 (SD 4.56) after 6 months (*P*<.001; 95% CI 1.04-1.48). Figure 2 shows MMD and MHD during the first 6 months of use after baseline.

AMD and migraine intensity also reduced from baseline to the sixth month after baseline (*P*<.001 for both). Headache intensity showed a numerically small, yet statistically significant increase from a mean 4.57 (SD 1.50) during baseline to a mean 4.71 (SD 1.85) in the sixth month after baseline (*P*<.001; 95% CI –0.19 to –0.06).

Subgroup analysis revealed a significant reduction in MMD, MHD, and AMD for both users with episodic and chronic headache in the sixth month after baseline. The slight increase in headache intensity remained significant only for users with episodic migraine, rising from a mean 4.77 (SD 1.47) during baseline to a mean 4.88 (SD 1.73) in the sixth month after baseline (*P*=.02; 95% CI –0.19 to –0.02), but not for users with chronic migraine. Headache characteristics for all patients, patients with episodic migraine, and patients with chronic migraine are shown in Table 2.

**Table 2:** Headache or migraine frequency and intensity, and use of acute medication during baseline at the third and the sixth month after baseline for all M-sense users, users with episodic migraine, and users with chronic migraine.

Outcomes		Baseline, mean (SD)	Month 6, mean (SD)	*P* value^a^	95% CI
**All M-sense users (N=1545)**					
	Monthly headache days	9.42 (5.81)	6.39 (5.09)	<.001	2.80 to 3.25
	Monthly migraine days	5.44 (4.98)	4.28 (4.56)	<.001	0.96 to 1.35
	Monthly days with acute medication use	5.62 (4.64)	4.35 (4.08)	<.001	1.08 to 1.45
	Mean monthly headache intensity	4.57 (1.50)	4.71 (1.85)	<.001	–0.19 to –0.06
	Mean monthly migraine intensity	5.42 (1.78)	5.22 (1.85)	<.001	0.09 to 0.27
**M-sense users with episodic migraine (n=985)**					
	Monthly headache days	8.23 (4.01)	5.63 (3.71)	<.001	2.35 to 2.85
	Monthly migraine days	5.64 (3.61)	4.38 (3.54)	<.001	1.04 to 1.48
	Monthly days with acute medication use	5.64 (4.43)	4.39 (3.91)	<.001	1.03 to 1.47
	Mean monthly headache intensity	4.77 (1.47)	4.88 (1.73)	.02	–0.19 to –0.02
	Mean monthly migraine intensity	5.36 (1.64)	5.19 (1.86)	.002	0.06 to 0.26
**M-sense users with chronic migraine (n=126)**					
	Monthly headache days	21.08 (4.90)	15.03 (7.03)	<.001	4.91 to 7.18
	Monthly migraine days	15.84 (6.02)	12.05 (7.21)	<.001	2.54 to 5.04
	Monthly days with acute medication use	8.79 (6.09)	6.71 (6.03)	<.001	1.29 to 2.85
	Mean monthly headache intensity	5.40 (1.69)	5.50 (1.90)	.21	–0.34 to 0.08
	Mean monthly migraine intensity	5.83 (1.46)	5.81 (1.72)	.22	–0.19 to 0.22

^a^*P* values as calculated with paired samples *t* test.

### Extended Analysis up to Month 12

For the extension up to 1 year, we analyzed the data of 812 users. Of the 559 users with available data, most (500/559, 89.4%) were female and on average 37.16 (SD 11.04) years old. The app algorithm supported the diagnosis of episodic migraine in 504 cases, chronic migraine in 68 cases, and another headache diagnosis in 240 cases.

**Figure 3:**
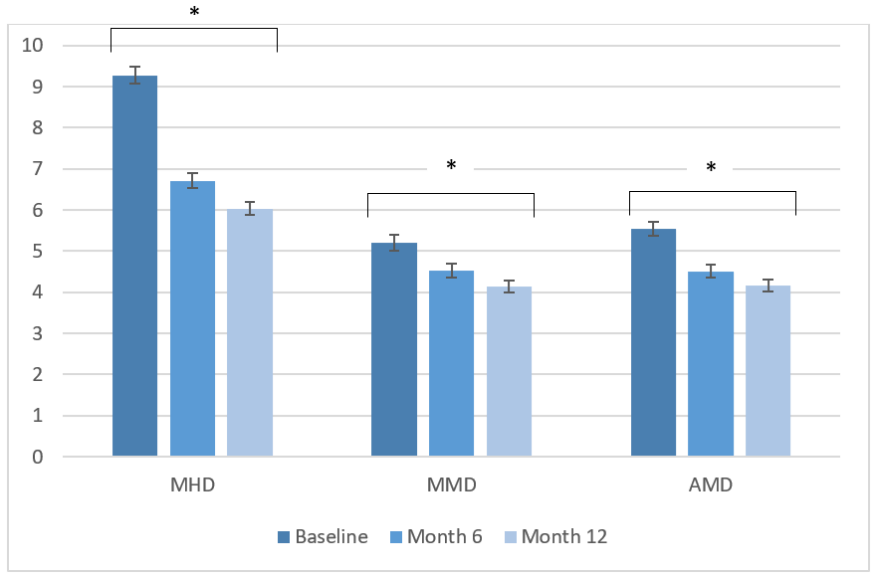
Monthly headache days, monthly migraine days, and monthly days with acute medication use (mean and SE) during baseline, and after 6 and 12 months of app use. Outcomes were compared using repeated-measures analysis of variance. AMD: days with acute medication use; HMD: monthly headache days; MMD: monthly migraine days .*P<.001.

These users reported a mean MHD of 9.27 (SD 5.88) during the baseline month, which gradually declined to 6.03 (SD 5.13) after 12 months (*P*<.001; 95% CI 2.90 to 3.57), as shown in Figure 3. MMD were reduced from a mean 5.20 (SD 4.95) during baseline to a mean 4.14 (SD 4.50) after 12 months (*P*<.001; 95% CI 0.79-1.33). Users also documented a decreased number of AMD (baseline: mean 5.54, SD 4.67; month 12: mean 4.16, SD 3.96; *P*<.001; 95% CI 1.19-1.64). Mean migraine intensity decreased significantly from baseline to month 12 (*P*=.004), while mean headache intensity was higher in month 12 (*P*=.02). Separate analyses for users with episodic and chronic migraine revealed similar results (Table 3).

**Table 3:** Headache and migraine frequency and intensity, and use of acute medication during baseline and the 12th month after baseline for all M-sense users, users with episodic migraine, and users with chronic migraine.

Outcomes		Baseline, mean (SD)	Month 12, mean (SD)	*P* value^a^	95% CI
**All M-sense users (N=812)**					
	Monthly headache days	9.27 (5.88)	6.03 (5.13)	<.001	2.90 to 3.57
	Monthly migraine days	5.20 (4.95)	4.14 (4.50)	<.001	0.79 to 1.33
	Monthly days with acute medication use	5.54 (4.67)	4.16 (3.96)	<.001	1.19 to 1.64
	Mean monthly headache intensity	4.40 (1.52)	4.66 (1.78)	.02	–0.24 to –0.03
	Mean monthly migraine intensity	5.33 (1.86)	5.11 (1.74)	.004	0.06 to 0.32
**M-sense users with episodic migraine (n=504)**					
	Monthly headache days	8.09 (4.03)	5.42 (3.77)	<.001	2.29 to 3.04
	Monthly migraine days	5.48 (3.53)	4.35 (3.69)	<.001	0.80 to 1.46
	Monthly days with acute medication use	5.65 (4.67)	4.31 (4.03)	<.001	0.99 to 1.70
	Mean monthly headache intensity	4.68 (1.52)	5.04 (1.74)	.26	–0.18 to 0.05
	Mean monthly migraine intensity	5.23 (1.86)	5.04 (1.74)	.02	0.03 to 0.34
**M-sense users with chronic migraine (n=68)**					
	Monthly headache days	21.31 (4.99)	14.21 (7.42)	<.001	5.28 to 8.92
	Monthly migraine days	15.38 (6.36)	11.06 (7.30)	<.001	2.65 to 5.99
	Monthly days with acute medication use	8.87 (5.63)	6.18 (5.17)	<.001	1.60 to 3.78
	Mean monthly headache intensity	5.32 (1.70)	5.44 (1.89)	.45	–0.41 to 0.18
	Mean monthly migraine intensity	5.80 (1.59)	5.82 (1.76)	.99	–0.28 to 0.28

^a^*P* values as calculated with paired samples *t* test.

## Discussion

We observed a significant improvement of headaches in patients who used the electronic headache diary on the M-sense app on a daily basis. The frequency of monthly migraine and headache days, days with use of acute medication, and migraine intensity declined over 6 months of daily app use. This decrease remained for regular users of the app after 12 months. These changes applied to the entire user population and to the subpopulations with episodic migraine and chronic migraine.

The observed changes in headache frequency are comparable to those of open-label studies with nonpharmacological treatments for headache prevention. Previous research on cognitive-behavioral treatment, relaxation training, or aerobic exercise in patients with primary headache disorders reported a reduction of 2 to 3 MHD after approximately 6 months of treatment, which is in the range of our analysis [18-20]. Of note, our population achieved this clinically meaningful improvement without any specific intervention apart from the basic M-sense app.

The main function of the basic version of the app is the electronic headache diary. The documentation of headache episodes in an app offers various advantages over a paper-and-pencil calendar. Most people have access to their smartphone at all times. This allows a more rapid “real-time” documentation in an app, while paper documentation is usually performed at a later time point and may be affected by a recall bias [3]. Backfilling of entries for several days is a common issue in paper calendars. In a study on chronic pain, 80 participants were asked to complete standardized pain assessments 3 times a day either in a paper or in an electronic calendar for 3 weeks [3]. The electronic diary was completed in 94% of cases within a predefined 30-minute time window, while this was the case for only 11% of entries in the paper calendar [3]. The group with the paper calendar documented about one-third of data after more than 1 day [3].

In our analysis, users documented headache information every day. Although completing diaries daily could be challenging [21], this allows for better data quality and validity. Completers of a daily internet-based migraine diary reported that it was a “major commitment but worthwhile” [22] and that it contributed to a better understanding of their headache disorder. An interactive visual presentation also facilitates data interpretation [23]. The graphic visualization of headache attacks in a monthly calendar may contribute to recognizing headache patterns over time and to raising self-awareness of the headache disorder [24]. Self-awareness in turn can lead to behavioral changes and a better treatment choice and adherence [25,26].

Another core function of the basic M-sense app is the recording of lifestyle factors that may trigger migraine attacks. The identification of individual trigger factors might also induce changes in behavior and influence the course of the headache disorders [27]. However, we cannot provide data on the number of individuals who used the trigger analysis on a regular basis. It was our primary goal to observe headache characteristics in users of a headache app in a real-world setting and not to analyze the single, specific functions. Therefore, we considered all offered app functions as part of a whole and not separately. Future research analyzing or comparing single app functions could provide further insights into the effectiveness of different features for different groups of users. Individual customization options might then enable users to adapt the app interface to their needs and obtain the largest possible benefits.

Comparisons between our analysis and other research on headache apps are only possible to a limited extent. In a review from 2016, Mosadeghi et al [28] identified only 6 studies about mobile apps for headache disorders. None of these studies assessed the change of headache characteristics during the use of a headache app and only evaluated the usability and feasibility of the apps through user surveys. In 2019, Göbel et al [29] reported results regarding the use of Migräne-App. Like M-sense, this app includes the documentation of headache information in a diary and the registration of weather data. Further features include a search function for headache experts, instructions for progressive muscle relaxation, a media library with educational resources, and an aura simulation. In contrast to M-sense, it does not record or analyze other lifestyle factors. Via an online survey, users of Migräne-App were questioned about their app use, their satisfaction with the app, and also about changes in headache patterns during the app use. Similar to our results, 1464 users reported a reduction of about 3 MHD and 1 AMD after using the app for more than 1 year. In contrast to our analysis, users manually entered their headache data from the app in an online survey, while we analyzed data directly from the app and therefore encountered a much lower risk of bias.

The main strengths of this analysis are the large sample size and the minimization of missing data, as we selected only users with daily app entries for up to 1 year. However, this could have led to a selection bias in favor of users who experienced noticeable benefits through the app, as users who experienced no benefit might have stopped using the app earlier. Furthermore, the daily use of an app also indicates a dedication to managing disease, which might have led to other preventive actions outside the use of the app. We had no further information about other pharmaceutical and nonpharmaceutical preventive therapies used by patients during the observation period that might have contributed to the change of headache frequency and intensity. We also cannot provide data about the frequency of app use in nondaily users or about how many users stopped using the app, as we received aggregated data only for the predefined population. The assessment of app-related risks was not within the scope of our analysis.

Another limitation concerns the classification of single headache attacks via the app algorithm. During app installation, users could indicate a pre-existing headache diagnosis. In users without a known headache diagnosis, TTH could be overrepresented because headache attacks with the characteristics of both probable migraine and TTH were classified by the algorithm as TTH according to the “general rule of hierarchy” in the ICHD-3 criteria [13]. However, in patients with a previous migraine diagnosis, these attacks were classified as migraine [14]. Further limitations relate to the definition of the episodic migraine and chronic migraine subgroups based on the classification by the app. To diagnose a headache disorder solely on the ground of the app data is not justified because characteristics and signs for other primary headaches or secondary headaches are not collected by M-sense and a physical examination is not included in the assessment. A definite headache diagnosis is only possible by the integration of the app data with the clinical assessment of a physician. Hence, we cannot rule out the possibility that some patients included in the study experienced headache disorders other than migraine or TTH.

Moreover, we included only those users who downloaded the basic version of M-sense. Other studies suggested a benefit of behavioral interventions through headache apps [30]. A multicenter, randomized controlled trial (SMARTGEM) is currently being conducted in Germany to test if the advanced active version of M-sense can confer a further advantage compared to the simple documentation of the basic version [31]. The first results are expected by the end of 2021. Through the Digital Healthcare Act (Digitale-Versorgung-Gesetz), physicians in Germany will be able to prescribe health care apps, and the costs will be reimbursed by statutory health insurance [32]. Therefore, evaluation of app-related health benefits will not only have clinical relevance but also major relevance in health economics.

In conclusion, our study found a decline in the mean values of headache and migraine characteristics after 6 months compared to baseline for people who regularly used an app as a headache diary. The reductions extended to 1 year for those who continued to regularly use the app. These results suggest that the regular use of an app to monitor headache and migraine characteristics may support standard headache care. Headache documentation in an app could help to raise awareness for headache disorders and to identify possible patterns and aggravating factors. It enables patients to take a more active role in managing their headache and feel more in control of their health, resulting in the improvement of headache over time. Based on our findings, the regular use of a headache app could represent an effective measure to complement the therapy of primary headaches.

## Abbreviations

AMD: days with acute medication use
ANOVA: analysis of variance
ICHD-3: The International Classification of Headache Disorders
MHD: monthly headache days
mHealth: mobile health
MMD: monthly migraine days
TTH: tension-type headache
